# Enhancing sleep quality in synucleinopathies through physical exercise

**DOI:** 10.3389/fncel.2025.1515922

**Published:** 2025-01-31

**Authors:** Jacopo Canonichesi, Laura Bellingacci, Francesco Rivelli, Alessandro Tozzi

**Affiliations:** Department of Medicine and Surgery, University of Perugia, Perugia, Italy

**Keywords:** neurodegeneration, non-pharmacological intervention, physical exercise, sleep disturbances, sleep hygiene, synucleinopathies

## Abstract

During sleep, several crucial processes for brain homeostasis occur, including the rearrangement of synaptic connections, which is essential for memory formation and updating. Sleep also facilitates the removal of neurotoxic waste products, the accumulation of which plays a key role in neurodegeneration. Various neural components and environmental factors regulate and influence the physiological transition between wakefulness and sleep. Disruptions in this complex system form the basis of sleep disorders, as commonly observed in synucleinopathies. Synucleinopathies are neurodegenerative disorders characterized by abnormal build-up of *α*-synuclein protein aggregates in the brain. This accumulation in different brain regions leads to a spectrum of clinical manifestations, including hypokinesia, cognitive impairment, psychiatric symptoms, and neurovegetative disturbances. Sleep disorders are highly prevalent in individuals with synucleinopathies, and they not only affect the overall well-being of patients but also directly contribute to disease severity and progression. Therefore, it is crucial to develop effective therapeutic strategies to improve sleep quality in these patients. Adequate sleep is vital for brain health, and the role of synucleinopathies in disrupting sleep patterns must be taken into account. In this context, it is essential to explore the role of physical exercise as a potential non-pharmacological intervention to manage sleep disorders in individuals with synucleinopathies. The current evidence on the efficacy of exercise programs to enhance sleep quality in this patient population is discussed.

## 1 Introduction

Synucleinopathies, including Parkinson’s disease (PD), dementia with Lewy bodies (LBD), and multiple system atrophy (MSA), are a group of neurodegenerative disorders characterized by the abnormal accumulation of *α*-synuclein (α-syn) protein aggregates in the brain. A variety of non-motor symptoms are encountered in synucleinopathies, and sleep disturbances are notably prevalent and significantly impact patients’ quality of life (Coon and Singer, 2020; Calabresi et al., 2023). Given the essential role of sleep in overall health and its influence on cognitive and physiological processes (Ramar et al., 2021), addressing sleep disturbances in individuals with synucleinopathies is a critical component of comprehensive care.

A fine-tuned and complex machinery regulates the processes that control the transition from sleep to wakefulness and vice versa, as well as the shifts between different stages of the sleep cycle. Various brain structures contribute to this regulation, and their function can be disrupted by *α*-syn aggregates, leading to the onset of sleep disorders in patients with synucleinopathies (Coon and Singer, 2020; Iranzo, 2016). On the other hand, poor sleep quality can exacerbate the motor, cognitive, and psychiatric manifestations of synucleinopathies, contributing to a decline in overall health. The existing literature on the necessity and quality of adequate sleep, the consequences of sleep deprivation, and the bidirectional relationship between synucleinopathies and sleep disorders is examined, with a focus on the possible pathophysiological mechanisms linking *α*-syn aggregates spreading and sleep–wake cycle disruption.

This review explores current evidence on how physical exercise can mitigate the pathophysiological mechanisms through which α-syn impacts sleep homeostasis. We discuss how physical activity supports the restoration of physiological circadian rhythms and induces adaptive responses that enhance protein aggregate clearance, promote neuroplasticity and neuronal survival, and regulate neuroinflammatory processes. Additionally, we examine how various forms of exercise therapy can alleviate the severity of sleep disorders in patients with synucleinopathies.

We conducted a literature review using PubMed,^1^ accessing articles published up to 2024 with the search terms “alpha-synuclein,” “exercise,” “sleep,” “synucleinopathy.” Together with a focus on preclinical studies linking *α*-syn aggregates accumulation and poor sleep quality, and on the possible mechanisms through which physical activity can help counteract α-syn-mediated damage, we reported experimental studies and metanalyses assessing the effects of different forms of physical exercise programs on outcomes associated with sleep quality, particularly in people suffering from PD.

## 2 Requirement of sleep and sleep quality

### 2.1 Sleep and brain health

Sleep is a fundamental physiological process orchestrated by the brain. It can be described as a phylogenetically conserved state characterized by limited movement and diminished responsiveness to environmental stimuli. While the expression of sleep varies across species, common features include the adoption of stereotyped postures and, crucially, its reversibility, which distinguishes it from states such as coma (Cirelli and Tononi, 2008).

Extensive research has been undertaken to elucidate the functions of sleep. Beyond its adaptive roles, significant evidence supports its critical involvement in cognitive processes, particularly the reorganization and consolidation of memories (Tononi et al., 2024; Tononi and Cirelli, 2014, 2020). Memory traces, when first formed, are fragile and susceptible to disruption by competing information encountered during wakefulness. Sleep has been hypothesized to facilitate the stabilization and integration of these memories. Studies demonstrate that information acquired before sleep, such as in the evening, is often better retained than information learned earlier in the day, highlighting the role of sleep in memory consolidation (Rasch and Born, 2013; Stickgold and Walker, 2013). Understanding the intricate relationship between sleep and memory remains a cornerstone of cognitive neuroscience, and disruptions of sleep cycle can lead to significant impairments in cognitive functions, emotional regulation, and physical health, underscoring the importance of maintaining good sleep hygiene and addressing sleep disorders promptly (Watson et al., 2015).

Although sleep is an indispensable physiological need for human health, with adults requiring a minimum of 7 h per day to promote optimal well-being (Hirshkowitz et al., 2015; Ramar et al., 2021; Watson et al., 2015), compelling economic and social pressures often lead to reductions in both sleep duration and quality.

Environmental factors such as noise, light pollution, and air pollution, alongside various social determinants, further hinder individuals from achieving a restorative amount of sleep (Rajaratnam and Arendt, 2001; Billings et al., 2020; Liew and Aung, 2021).

Numerous factors play a pivotal role influencing the individuals’ time spent on sleep, including genetics, medical conditions, behaviors, and environmental influences. Inadequate sleep quantity is associated with a myriad of adverse consequences, including impaired cognitive function, and long-term detrimental effects on cardiovascular, immune, and metabolic health. Collectively, these issues constitute the constellation of symptoms enclosed in the “insufficient sleep syndrome” (American Academy of Sleep Medicine, 2023). Focusing on the relationship between sleep and brain health, insufficient sleep negatively impacts synaptic function and brain network dynamics. Sleep deprivation has been seen to affect hippocampal long-term potentiation and adult neurogenesis, as well as to have repercussions on hippocampal connectivity and subsequent memory encoding (Krause et al., 2017). Reduced sleep time has also been seen to be associated with altered activity in brain regions involved in attention, emotional processing and goal-oriented behavior, such as dorsolateral prefrontal cortex, thalamus and basal ganglia (Krause et al., 2017). In addition, disturbed sleep has been suggested as a possible modifiable risk factor for cognitive impairment and dementia (Ju et al., 2014; Livingston et al., 2017; Minakawa et al., 2019). Accordingly, interventions aimed at improving sleep quality have been addressed as potential secondary prevention strategies for dementia (Ju et al., 2014; Livingston et al., 2017; Irwin and Vitiello, 2019).

Since sleep is highly relevant for memory consolidation, it has been hypothesized that a preserved sleep architecture might be determinant for the development of compensatory mechanisms following the loss of physiological brain circuits structure, contributing to preserving cognitive functions (Sanchez et al., 2022; Kanishka and Jha, 2023). Therapeutical and experimental approaches, primarily aimed at improving sleep, were proven to promote adaptive modifications of brain circuits and compensate for damage to brain structures, particularly to those involved in critical functions such as memory, motor control, or sensory processing. For instance, blue light exposure used to reset circadian clock in people recovering from traumatic brain injury (TBI) was shown to enhance thalamo-cortical connectivity and increase posterior thalamus volume, together with improving sleep–wake rhythm (Killgore et al., 2020). Furthermore, a more preserved sleep architecture after TBI was reported to correlate with better cognitive performance over time (Sanchez et al., 2022). Similar to what is reported for TBI, sleep also contributes to adaptive neuroplastic processes and functional recovery after stroke (Duss et al., 2017). Moreover, optogenetic induction of sleep slow wave oscillatory activity promoted axonal sprouting at the peri-infarctual zone and determined a better motor recovery in a murine model of stroke (Facchin et al., 2020).

### 2.2 Sleep quality measurements

While the number of sleep hours is critical, the qualitative aspects of sleep are equally significant in determining its restorative efficacy (Buysse, 2014; Kohyama et al., 2018; Kohyama, 2021). Assessing sleep quality can pose challenges, and no definitive methods exist. The Pittsburgh Sleep Quality Index (PSQI), developed in 1988, is one of the most widely used scales for evaluating qualitative sleep aspects. It comprises 19 questions pertaining to sleep habits over the past month, encompassing subjective sleep quality, sleep onset latency, sleep duration, habitual sleep efficiency, sleep disturbances, use of sleep medication, and daytime dysfunction. Each aspect is assigned a specific score, culminating in an overall sleep quality score (Buysse et al., 1989).

Distinctions need to be made between objective and subjective sleep quality. Objective measures, obtained through techniques like polysomnography, assess physiological parameters such as brain activity, respiration, eye movements, and heart rate during sleep. Polysomnography is particularly valuable for identifying sleep disorders. However, objective data may not always align with an individual’s subjective perception of sleep quality upon waking. Subjective assessments, such as those provided by scales like the PSQI, put an emphasis on feelings of refreshment upon waking and daytime mental state following sleep, recognized as crucial determinants of subjective sleep quality (Kohyama, 2021).

In brief, both quality and quantity of sleep are essential factors in ensuring the well-being of the body and its proper functioning. As recently reported in a position paper by the American Academy of Sleep Medicine (Ramar et al., 2021), it is crucial for general population to acquire this awareness and prioritize sleep in their lives to prevent a wide range of health problems.

## 3 Sleep imbalance in synucleinopathies

### 3.1 Neurodegeneration and sleep disruption

Inadequate or disrupted sleep is closely linked to various adverse health conditions, particularly neurodegenerative disorders. Although the relationship between sleep and neurodegenerative diseases is likely reciprocal, sleep disturbances often emerge before other key clinical symptoms. During sleep, the elimination of neurotoxic waste in the brain, in particular through the activity of the glymphatic system, is significantly enhanced, accordingly, sleep deprivation negatively affects the clearance of misfolded proteins, leading to the build-up of pathological aggregates implicated in neurodegenerative diseases (Nedergaard and Goldman, 2020; Xie et al., 2013). Among the amyloidogenic proteins that can go through misfolding and accumulation in neurodegenerative diseases, are the amyloid beta (Aβ), tau, or *α*-syn. While the presence of these aggregated protein fibrils is a defining feature of neurodegenerative disorders, smaller, soluble toxic oligomers of these proteins are believed to form earlier and may contribute to disease progression before the appearance of larger aggregates (Rowe et al., 2024).

Reduction of sleep hours and sleep fragmentation directly sustain neuronal damage in neurological disorders in which proteinopathy is a key hallmark, such as Alzheimer’s disease (AD) or PD. Moreover, experimental evidence showed that disrupted circadian rhythm is also associated with increased oxidative damage and altered inflammatory response, both factors that take part to neurodegeneration and have a crucial role in the pathophysiology of these diseases (Musiek and Holtzman, 2016; Leng et al., 2019; Irwin and Vitiello, 2019). Sleep disorders are commonly assessed as comorbidities in neurodegenerative disorders (Wulff et al., 2010; Livingston et al., 2017), and together with contributing to altered amyloidogenic molecules turnover, brain network alterations secondary to reduced sleep time might take part to the clinical manifestations observed (Minakawa et al., 2019).

Conversely, pathological processes involved in cognitive deterioration can also affect sleep. For instance, Aβ and *α*-syn were proven to affect circadian clock genes transcription patterns (Musiek and Holtzman, 2016), and experimentally-induced alterations in neuronal mechanisms of memory consolidation have been reported to reverberate on sleep architecture (Kumar and Jha, 2017). Additionally, brain areas controlling sleep appear to be susceptible to degenerative processes as it can be assessed in the context of different neurological and neuropsychiatric disorders (Wulff et al., 2010; Benca and Teodorescu, 2019).

Overall, on one hand sleep deprivation has been addressed as a trigger for pathophysiological processes responsible for neurodegeneration, and a contributing factor to functional alterations underlying cognitive impairment; on the other hand, mechanisms responsible for cognitive impairment can also affect sleep–wake regulation, favoring the occurrence of sleep disorders. The relationship between neurodegenerative processes and disturbed sleep is particularly evident in a group of diseases characterized by the formation and accumulation of *α*-syn aggregates, referred as synucleinopathies.

### 3.2 Sleep disorders in synucleinopathies: possible causal mechanisms

*α*-Syn is a small protein expressed at the synaptic level and in white blood cells, that presents a disorganized structure and it is capable, under physiological conditions, to fold and form tetrameric structures. Under pathological conditions, folded *α*-syn can form higher molecular weight structures, up to the formation of intracellular insoluble inclusions (Calabresi et al., 2023).

A broad spectrum of clinical features can be observed in the context of synucleinopathies, ranging from hypokinetic disorders to dementia and psychiatric symptoms, and these different manifestations accompany distinct patterns in *α*-syn aggregates spreading throughout the brain, involving brainstem nuclei, basal ganglia, and cortical areas. Features that accompany, at a varying degree, cognitive and motor manifestations, are sleep disturbances and autonomic dysfunction, characterized by altered thermoregulation, sweating, gastrointestinal symptoms, bladder disturbances, and orthostatic hypotension. Of note, also autonomic dysfunction can exist as the only manifestation of synucleinopathy, taking the name of pure autonomic failure (PAF). Similarly, REM sleep behavior disorder (RBD), a parasomnia characterized by the loss of muscle atonia during REM sleep, leading to the enactment of dreams and potential injury to oneself or bed partner, has been seen to exist as a nosological entity *per se* associated to *α*-syn aggregates accumulation (Coon and Singer, 2020).

The spreading of α-syn aggregates plays a pivotal role in the pathogenesis of synucleinopathies, including PD and MSA. *α*-Syn pathology is characterized by the accumulation of misfolded α-syn protein aggregates, which propagate in a prion-like manner throughout the nervous system, contributing to neuronal dysfunction and degeneration (Tozzi et al., 2021). This propagation occurs through the intercellular transfer of *α*-syn aggregates between interconnected neurons, leading to the progressive spread of pathology (Olanow and Brundin, 2013; Goedert et al., 2017). At the cellular level, *α*-syn aggregates disrupt synaptic function and neurotransmitter release, impairing neuronal communication (Tozzi et al., 2021). α-Syn oligomers directly interact with synaptic vesicles and interfere with their trafficking and fusion, leading to synaptic dysfunction (Burré, 2015; Lashuel et al., 2013). Furthermore, α-syn aggregates induce neuroinflammation and oxidative stress, further exacerbating neuronal damage and dysfunction (Gao et al., 2011; Hirsch et al., 2005).

Animal models of synucleinopathy, including the *α*-syn pre-formed fibril (PFF) rat model, provide valuable insights into how α-syn aggregation influences neuronal circuits and neurotransmitter systems (Tozzi et al., 2016, 2021). By elucidating the early molecular and cellular changes associated with *α*-syn pathology in the PFF rat model, researchers can pinpoint potential therapeutic targets to ameliorate sleep patterns in synucleinopathies.

Several neurotransmitter systems and their associated signaling pathways are affected by the spreading of *α*-syn aggregates. In PD, dopaminergic neurons within the substantia nigra pars compacta (SNpc) are particularly vulnerable to α-syn pathology. The loss of dopaminergic neurotransmission in the striatum results in the characteristic motor symptoms of PD, such as bradykinesia, rigidity, and tremor (Braak et al., 2003; Surmeier et al., 2017).

The degeneration of the dopamine signaling system, mediated by the propagation of *α*-syn aggregates, profoundly influences sleep quality and the sleep–wake rhythm, as it can be observed in PD. Dopamine plays a crucial role in the regulation of sleep–wake patterns through its involvement in the maintenance of arousal and modulation of the transition from NREM to REM phases during sleep cycle (Dauvilliers et al., 2015; Lima, 2013). In experimental PD, the progressive loss of dopaminergic neurons in the SNpc was shown to contribute to the development of sleep–wake rhythm abnormalities (Fifel and Cooper, 2014).

In addition to dopaminergic dysfunction, other neurotransmitter systems are also impacted by *α*-syn pathology. Serotonergic, noradrenergic, and cholinergic neurons are affected in PD and MSA, contributing to non-motor symptoms such as cognitive impairment, autonomic dysfunction, and mood disorders (Halliday et al., 2014; Stefanova et al., 2009; Kalia and Lang, 2015; Tozzi et al., 2016; Buddhala et al., 2015; Valli et al., 2022).

Several other mechanisms underlie the impact of α-syn pathology on sleep regulation. Firstly, α-syn aggregates accumulate in various brain regions involved in sleep regulation, including the hypothalamus, brainstem, and basal forebrain, disrupting their normal function (Iranzo, 2013; French and Muthusamy, 2018). Specifically, α-syn aggregates may directly affect sleep-promoting neurons in the ventrolateral preoptic nucleus and the orexin-producing neurons in the hypothalamus, further exacerbating sleep disturbances (Fronczek et al., 2007). Furthermore, neuroinflammation and synaptic dysfunction induced by α-syn aggregation (Calabresi et al., 2023) may exacerbate sleep disturbances by altering neuronal connectivity and neurotransmission (Zielinski and Gibbons, 2022).

A bidirectional relationship links synucleinopathies, circadian rhythm disturbances, and sleep disorders. As stated earlier, unfolded protein clearance is less effective in case of insufficient sleep. On the other hand, α-syn aggregates accumulation can contribute to the onset of sleep disorders, with RBD as the most paradigmatic example. Indeed, the development of RBD is a strong predictor of the development of PD and DLB (Iranzo, 2013; Peever and Fuller, 2017; Dauvilliers et al., 2018; Leng et al., 2019). Also, other sleep disturbances are associated with synucleinopathies, comprising insomnia, restless legs syndrome, and circadian rhythm sleep–wake disorders (Chahine et al., 2017).

## 4 Sleep quality improvement in synucleinopathies

In the broad spectrum of non-motor manifestations observed in synucleinopathies, particularly in PD, sleep disturbances still lack consensus on effective pharmacological and non-pharmacological interventions. This is partly due to the lack of dedicated studies on the topic, resulting in most currently used treatments relying on weak clinical evidence. According to the most recent recommendations from “The International Parkinson and Movement Disorder Society Evidence-Based Medicine Committee,” medications classified as possibly useful for treating insomnia include the dopaminergic agonist rotigotine, the prohypnotic drug eszopiclone, and melatonin. For excessive daytime sleepiness, modafinil is classified as possibly useful (Seppi et al., 2019). Non-pharmacological interventions have also been reported to benefit sleep disorders in the context of synucleinopathies. Positive pressure ventilation has been shown to effectively improve both insomnia symptoms and daytime sleepiness, particularly in cases associated with obstructive sleep apnea (Seppi et al., 2019).

Cognitive-behavioral therapy (CBT) programs have also been reported to improve both objective parameters and self-perceived sleep quality in PD patients suffering from insomnia. However, small group sizes and the lack of standardized treatment protocols prevent the definition of a universally applicable therapeutic strategy (Patel et al., 2017; Humbert et al., 2017; Lebrun et al., 2020).

Looking ahead, a deeper understanding of the pathophysiological mechanisms underlying sleep–wake cycle alterations in synucleinopathies is paving the way for treatment strategies. These strategies may involve Zeitgebers, such as light exposure, or the identification of novel molecular targets involved in the regulation of circadian rhythms (Feigl et al., 2024).

Although there is still no definitive management strategy for sleep disorders associated with synucleinopathies, different lines of both preclinical and clinical evidence highlight the benefits of an improved sleep quality specifically in these patients. For instance, a retrospective study of PD patients demonstrated a negative correlation between objective sleep quality and motor progression, indicating that more restorative sleep can positively affect the disease’s natural course (Schreiner et al., 2019). Conversely, sleep fragmentation has been linked to more severe dopaminergic neuron loss and a higher likelihood of a pathological PD diagnosis (Sohail et al., 2017). Sustaining the hypothesis that restoring sleep architecture might contribute to ease other clinical aspects of PD, a recent double-blinded randomized trial showed a positive correlation between sleep outcomes improvement after transcranial magnetic stimulation and motor function. Indeed, repetitive stimulation at the right dorsolateral prefrontal cortex of people with PD was proven effective to ameliorate subjective sleep quality and sleep architecture, together with reducing motor symptoms severity (Wu et al., 2024).

Considering the bidirectional relationship between impaired clearance of misfolded proteins and non-restorative sleep, it has been suggested that restoring a physiological sleep–wake cycle can enhance the efficiency of protein clearance systems and reduce oxidative stress, potentially slowing pathological progression (Yang et al., 2020). Interestingly, studies have shown that a nuclear magnetic resonance index, estimating glymphatic system activity, is generally lower in cases of possible RBD and PD compared to healthy controls. Moreover, a negative correlation exists between glymphatic system function and the severity of RBD (Si et al., 2022). Thus, beyond the benefits to the overall quality of life for patients and their caregivers, improving sleep quality may also have disease-modifying potential. For this reason, it is crucial to identify effective, widely applicable, and low-risk therapeutic strategies for sleep disorders associated with synucleinopathies.

## 5 Exercise therapy in sleep hygiene: a focus on synucleinopathies

### 5.1 Exercise as a non-pharmacological intervention in neurodegenerative disorders

Numerous studies have demonstrated that physical exercise can serve as an effective therapeutic tool for individuals with neurodegenerative disorders, helping to slow down pathological processes affecting the central nervous system (CNS). Accordingly, the benefits of physical activity have been demonstrated in various models of neurodegenerative disorders (Lee et al., 2021), whereas sedentary behavior has been shown to exacerbate disability in individuals with chronic neurological conditions such as PD and multiple sclerosis (Ellis and Motl, 2013). Furthermore, sedentariness may represent a potential risk factor for developing all-cause dementia (Raichlen et al., 2023). A key benefit of physical activity lies in its ability to upregulate neurotrophin pathways, particularly brain-derived neurotrophic factor (BDNF), within the CNS. This effect is mediated by the peripheral release of irisin, as highlighted by recent findings (Lee et al., 2021; Reddy et al., 2023; Walzik et al., 2024). Enhanced neurotrophins signaling has been addressed as a key mechanism through which physical exercise contributes to brain health, promoting neuronal survival and neuroplasticity, ultimately contributing to cognitive function preservation (Hötting and Röder, 2013; Mandolesi et al., 2018). Intriguingly, BDNF signaling has also been seen to downregulate the production of the amyloidogenic peptide Aβ (Rohe et al., 2009; Nigam et al., 2017); conversely, defective BDNF signaling has been reported to favor intracellular *α*-syn aggregates formation in the SNpc of aged mice (Von Bohlen und Halbach et al., 2005). BDNF was also shown to counteract dopaminergic degeneration in experimental PD (Sun et al., 2005). Of note, both preclinical evidence (Yuan et al., 2010; Fang et al., 2017) and pathological findings in brains from people suffering from PD (Kang et al., 2017) demonstrate that BDNF signaling is impaired by pathological α-syn accumulation. By favoring BDNF synthesis, physical activity might counterbalance the effects of aggregated α-syn, and slow down neurodegenerative processes. In line with this assumption, a recent study utilizing the PFF rat model of synucleinopathy has demonstrated that vigorous and consistent physical activity can decelerate the progression of both non-motor and motor symptoms of PD. This effect is achieved by reinstating activity-dependent long-term plasticity through the enhancement of BDNF pathway (Marino et al., 2023).

Moreover, physical exercise might contribute to the clearance of amyloidogenic peptides and proteins from the CNS also through the enhancement of glymphatic system activity, both indirectly as a consequence of a better cardiovascular health, and directly by upregulating the polarized expression of aquaporin at astrocytes endfeet (Formolo et al., 2023; Olegário et al., 2024). Additionally, studies on murine models of AD and PD subjected to forced physical activity showed reduced astrogliosis and microgliosis, along with increased expression of anti-inflammatory cytokines and decreased expression of proinflammatory cytokines, compared to sedentary controls (Sun et al., 2018; Liu et al., 2020; Leem et al., 2023).

Consistent with these findings, cumulating clinical evidence highlights that different forms of physical activity can effectively alleviate clinical manifestations of synucleinopathies. Different exercise-based therapeutic approaches have been attempted in people with PD, ranging from cardiovascular, resistance, and strength training, as well as traditional disciplines such as yoga, tai-chi, dance, occupational therapy and multicomponent exercise programs (Yang et al., 2022; Ernst et al., 2023). Consequently, systematic reviews and network metanalyses have been conducted to organize and categorize these diverse interventions, as well as to establish which exercise programs were seen to better counteract motor and non-motor manifestations of PD (Yang et al., 2022; Ernst et al., 2023). The outcomes of these studies are summarized in Table 1. Furthermore, the enrolment of PD patients in remotely supervised physical activity has been shown to have a positive impact on functional connectivity in brain regions implied in motor control, and to be associated with a significant reduction in the degree of global brain atrophy over time (Johansson et al., 2022).

**Table 1:** Effects of physical exercise programs in PD and experimental synucleinopathy.

Author	Study type	Objectives	Subjects	Sample size	Type of exercise	Main findings	Main limitations
Elyazed et al. (2018)*	RCT	Evaluation of PE as a therapeutic strategy to counteract insomnia and depressive symptoms in PD	65–70-year-old adults with PD	15 AE + physical therapy vs. 15 physical therapy	AE	Reduction of insomnia severity index	No information about clinical stage of PD and concomitant therapeutic interventions
van der Kolk et al. (2019)*	RCT	Assessment of home-based PE intervention as a nonpharmacological treatment to counteract motor manifestations severity in PD	Park-in-Shape trial: 30-75-year-old adults with PD. Hoehn & Yahr stage ≤2	65 AE vs. 65 stretching	AE	Amelioration of off-state motor manifestations severity	Adherence to PE programs was assessed through questionnaires
Amara et al. (2020)*	RCT	Evaluation of the effects of RT on objective sleep quality measurements	≥45-year-old adults with PD, Hoehn & Yahr stage 2–3, not in a regular PE program	27 RT vs. 28 no-exercise	RT	Increased total sleep time and sleep efficiency, prolonged SWS duration. Enhanced subjective sleep quality assessed through PSQI, not observed by applying Epworth sleepiness scale	PSG assessment performed only on one night for each time point
Cristini et al. (2021)	MA	Assessment of sleep quality improvement in people with PD undergoing PE programs	≥18-year-old adults with PD	12 studies (690 people)	ST, RT, AE, BT, TMD, dance	Overall improvement of subjective sleep quality	Overall effect was evaluated by considering different exercise protocols altogether
Johansson et al. (2022)	RCT	Evaluation of neural mechanisms underlying motor control improvement in people with PD undergoing AE	People recruited in the Park-in-Shape trial (as in van der Kolk et al., 2019)	23 AE vs. 31 stretching	AE	Increased cortico-putaminal and right frontoparietal network connectivity, reduced brain atrophy and enhanced motor control	Adherence to PE programs was assessed through questionnaires
Yang et al. (2022)	MA	Evaluation of the effectiveness of different types of PE interventions on attenuating motor and nonmotor manifestations in people with PD	People diagnosed with PD	250 studies (13,011 people)10 studies (452 people) reporting sleep quality measurements	ST, RT, AE, BT MCE, TMD, AQE, dance	Benefits from different PE programs on motor symptoms, muscle strength, anxiety, depression and cognitive functionSleep quality improvement in people undergone RT (2 trials), no significant improvement with other exercise protocols (8 trials)	Takes no consideration of the training period duration and activity intensity
Ernst et al. (2023)	MA	Individuation of which type of PE programs give the greatest benefits on motor manifestations and quality of life in people with PD	People diagnosed with PD	156 studies (7,939 people)	ST, RT, ET, AE, BT MCE, TMD, AQE, dance, gaming	Moderate benefit from dance (high confidence), AQE, BT and MCE (low confidence) on motor signs; Large benefit from AQE (high confidence), moderate benefit from ET and small from BT and MCE (low confidence) on overall quality of life	
Marino et al. (2023)	PS	Evaluation of the capacity of PE to counteract pathological and clinical progression in synucleinopathies	Rat model of synucleinopathy obtained through intrastriatal PFF injection (PFF rat)	29 AE trained PFF rats vs. 20 sedentary PFF rats vs. 30 AE trained controls vs. 29 sedentary controls	AE	Attenuated dopaminergic degeneration, rescue of striatal neurons plasticity and dendritic spine density, improved motor and cognitive performance	

Selected evidence from experimental studies on the effectiveness of different physical exercise programs in ameliorating clinical and pathological aspects of PD and experimental synucleinopathy. AE, aerobic exercise; AQE, aquatic exercise; BT, Balance training; MCE, multicomponent exercise; PE, physical exercise; PSG, polysomnographic; RT, resistance training; RCT, randomized control trial; ST, strength training; TMD, traditional movement disciplines, comprising Yoga, Qigong, and Tai Chi. *These studies are also included in the metanalysis from Cristini et al. (2021) for the evaluation of subjective sleep quality improvement.

### 5.2 Effects of exercise on sleep architecture

Numerous studies have suggested that physical activity can have beneficial effects on both the quantity and quality of sleep, as well as be a suitable strategy to improve the symptoms associated with sleep disorders (Banno et al., 2018; Hasan et al., 2022). Moreover, physical exercise can also be included as an addition to other therapeutic strategies. Different physical exercise programs were reported to be feasibly applicable in combination with CBT in adults suffering from anxiety disorders, with no significant impact on treatment adherence (Frederiksen et al., 2021). The combination of exercise therapy to CBT has also been proposed for treating insomnia, although evidence on the benefits of combining the two approaches to improve sleep outcomes is still lacking (Passos et al., 2023).

Physical activity can serve as an ally for sleep hygiene, acting as a Zeitgeber and contributing to a better perception of rest during sleeping hours. Among the mechanisms through which exercise can promote sleep is the effect of physical activity on the regulation of body temperature. Indeed, the modulation of core body temperature due to acute physical activity, along with exercise’s role in resetting circadian metabolic fluctuations in muscle tissue and melatonin secretion patterns, can facilitate the induction of sleep and the maintenance of a physiological sleep–wake cycle (Driver and Taylor, 2000; Atkinson et al., 2007; Escames et al., 2012; Gabriel and Zierath, 2019). Experimental evidence highlights the profound influence of physical activity on circadian rhythms, modulating the expression patterns of core clock genes. Depending on the type, duration, intensity, and timing of exercise, it can induce either an advancement or a delay in core body temperature and melatonin rhythms (Shen et al., 2023).

Furthermore, physical activity can affect the release of neurotransmitters implicated in the central modulation of sleep–wake cycle, such as serotonin (Mistlberger et al., 2000; Otsuka et al., 2016). Notably, physical activity might also be sufficient to restore serotonin levels under pathological conditions, as observed in a model of vascular dementia (Zhang et al., 2020; Fan et al., 2022). Similar to serotonin, also *γ*-aminobutyric acid (GABA), the main inhibitory neurotransmitter in the brain, is implied in the central modulation of wakefulness and sleep, being its receptors the main pharmacological target for traditional pro-hypnotic drugs (Gottesmann, 2002). CNS levels of GABA were seen to decrease with aging, and the disruption of GABAergic system is deemed to contribute to the pathogenesis of several neurological disorders (Novak et al., 2024; Zuppichini et al., 2024). Evidence from magnetic resonance spectroscopy has shown that physical exercise can acutely increase brain GABA levels in humans (Novak et al., 2024). Accordingly, transcranial magnetic stimulation studies show that exercise can modulate cortical excitability both acutely and chronically, giving indirect evidence on the capacity of physical activity to influence GABAergic system (Novak et al., 2024).

Aside from the possible beneficial effects of exercise on sleep quality and quantity due to rapid neuroendocrine adaptations, regularly performed physical activity has been shown to improve sleep quality in both good sleepers and in people suffering from sleep disorders. Moreover, the positiveness of physical activity on underlying pathology and general health should be considered. In poor sleepers, exercise therapy has been seen to impact the self-perceived sleep quality and to reduce of insomnia severity (Driver and Taylor, 2000; Banno et al., 2018). Regular exercise has been shown to improve sleep continuity and architecture, resulting in decreased sleep latency and increased slow wave sleep (SWS) duration (Kredlow et al., 2015). In particular, several suitable therapeutic schemes have been successfully applied in elderly people, ameliorating sleep quality, as shown in a recent systematic review and network metanalysis (Hasan et al., 2022). In this work, endurance training combined with walking demonstrated the highest benefit on self-perceived sleep quality among different kinds of exercise therapy, (88.9% of surface under the cumulative ranking curve value). Noteworthy, CBT showed even greater efficacy (99.9%), proving to be effective in all the trials included in the metanalysis. Notably, walking was reported to give benefits also in people with neurocognitive comorbidities (Hasan et al., 2022). Significant improvements were also highlighted for other exercise therapy strategies, such as tai-chi, resistance training, yoga, or Nordic walking (Hasan et al., 2022).

Non-motor manifestations, and in particular sleep disorders, pose challenges in the management of patients with synucleinopathies, due to a limited understanding of their underlying pathophysiological mechanisms and the limited efficacy of pharmacological treatments (Taximaimaiti et al., 2021). Consequently, there is growing interest in developing non-pharmacological strategies to alleviate sleep disturbances associated with synucleinopathies. Emerging evidence suggests that regular exercise exerts beneficial effects on sleep quality and sleep-related outcomes in various clinical populations, including individuals with cognitive impairment (Hasan et al., 2022).

### 5.3 Exercise therapy to improve sleep in synucleinopathies

In synucleinopathies, exercise therapy holds promise as a non-pharmacological intervention for ameliorating sleep disturbances and enhancing overall sleep health. Regular physical activity has been proven overall beneficial in people suffering from PD by acting on several independent factors, ranging from general well-being due to effects on cardiovascular and osteo-skeletal systems, as well as a positive impact on autonomic functions and neuroplasticity (Speelman et al., 2011; Petzinger et al., 2013).

Regarding specifically PD-associated sleep disorders, a recent systematic review and metanalysis has demonstrated an overall improvement of referred sleep quality in PD patients regularly performing exercise therapy at an intensity that was sufficient to increase metabolic activity in a measurable way—such as an increase of heart rate—twice a week or more for at least four weeks (Cristini et al., 2021). This improvement was also assessed by objective measures obtained through polysomnography in a randomized controlled trial (RCT) conducted by Amara and colleagues (Amara et al., 2020). The study highlighted a statistically significant amelioration of objective sleep quality measurements. During the course of the study, people with PD underwent supervised 16-week resistance training and functional mobility exercises, three days per week. Compared to the control group which underwent sleep hygiene interventions, the exercise therapy group showed a statistically significant prolongation of total sleep time (an average increase of 30 min vs. an average decrease of 15 min in the sleep hygiene control group) and a relatively longer SWS duration (+5% vs. +1% of total sleep time) (Amara et al., 2020). Furthermore, a study included in the metanalysis from Cristini et al. (2021) showed an improvement in self-perceived insomnia severity in PD patients performing moderate intensity physical therapy and cardiovascular training three times a week. This study demonstrated a statistically significant reduction in the Insomnia Severity Index following exercise therapy (Elyazed et al., 2018; Cristini et al., 2021). More recently, an attempt has been made to define and standardize the best approach for introducing exercise therapy as a tool to improve sleep quality in the course of PD, through a single-blind randomized controlled trial (Cristini et al., 2024).

Designing an optimal exercise prescription for individuals with synucleinopathies requires consideration of various factors, including disease severity, functional status, comorbidities, and individual preferences. Current guidelines recommend a multimodal approach incorporating aerobic, resistance, and flexibility exercises tailored to the specific needs and capabilities of each patient (American College of Sports Medicine et al., 2022). Aerobic exercises, such as walking, cycling, and swimming, are particularly beneficial for improving cardiovascular fitness, enhancing mood, and reducing depressive symptoms (Reid et al., 2010), which are commonly associated with synucleinopathies (Stefanova et al., 2009; Kalia and Lang, 2015; Taylor et al., 2020). Furthermore, exercise programs including tai chi can improve individual balance, while flexibility exercises can alleviate muscle stiffness and enhance joint mobility (American College of Sports Medicine et al., 2022; Yang et al., 2022) contributing to overall well-being and potentially ameliorating sleep quality.

## 6 Conclusion

**Figure 1:**
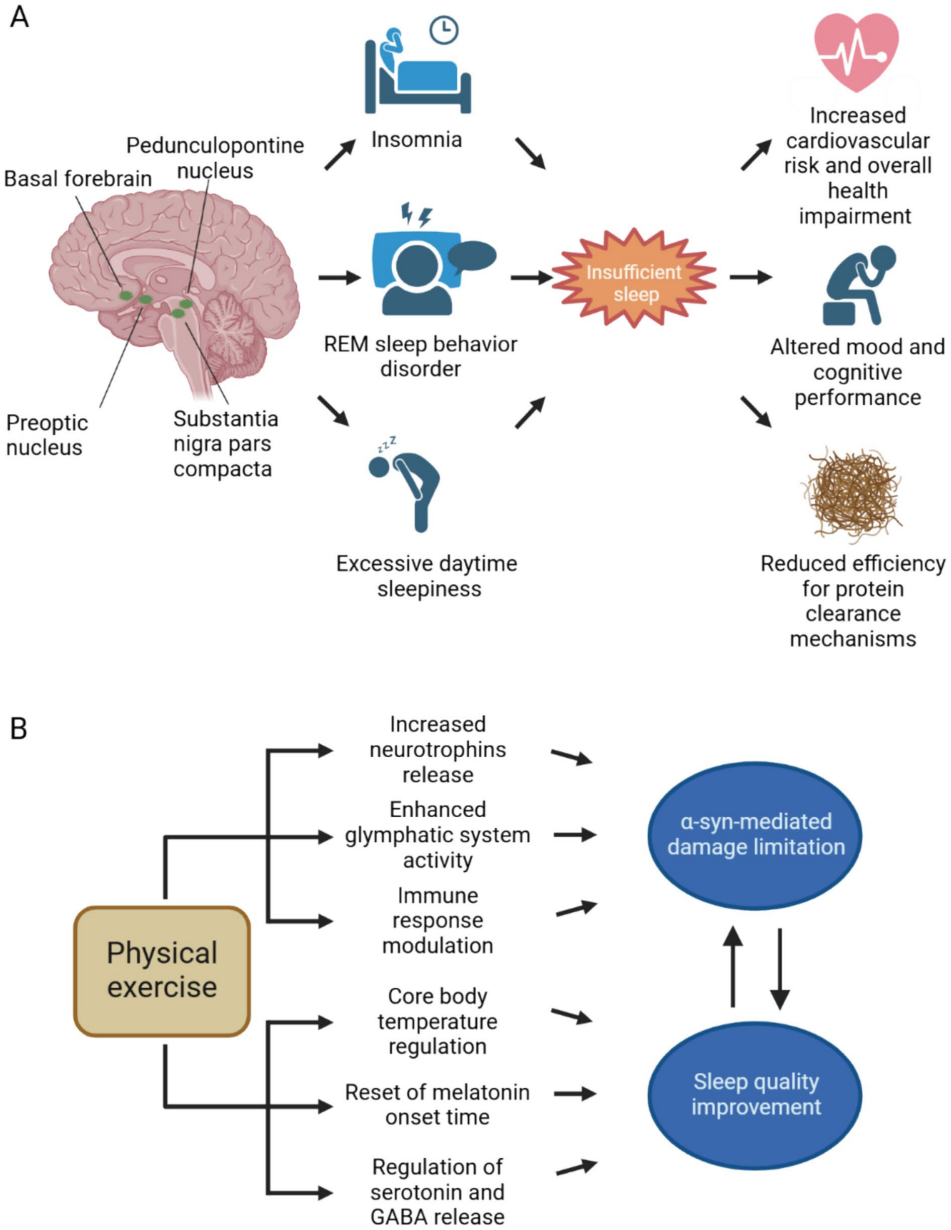
Neuroanatomical bases of sleep disorders in synucleinopathies and therapeutical effects of physical exercise. **(A)** The spreading of aggregated α-syn involves different brain areas implied in both circadian rhythm and sleep cycle regulation. Both substantia nigra pars compacta and pedunculopontine nucleus, through dopaminergic and cholinergic neurotransmission, respectively, regulate the arousal state during wakefulness and sleep. Also, the hypothalamus and the basal forebrain are affected by α-syn spreading. Sleep disturbances, such as insomnia, REM sleep behavior disorder and excessive daytime sleepiness are commonly encountered in the clinical practice in patients with synucleinopathies. Insufficient sleep contributes to increased cardiovascular risk and negatively impacts on overall health, mood and cognition. Since sleep is crucial for effective neurotoxic waste clearance, insufficient sleep may further contribute to α-syn spreading and subsequent neurodegeneration. **(B)** Physical exercise counteracts α-syn accumulation and its neurotoxic effects, by promoting neurotrophins release and extracellular space clearance, furthermore it contributes to the resolution of neuroinflammatory response by promoting anti-inflammatory cytokines secretion. By acting against α-syn mediated damage, physical activity can ultimately improve sleep. Through the reset of circadian rhythm and the modulation of neurotransmitter systems implied in sleep–wake cycle, exercise can increase sleep duration and enhance sleep quality. Better sleep quality, on the other hand, slows down pathological progression in synucleinopathies. Abbreviations: GABA, γ-aminobutyric acid; REM, rapid eye movement. Created with BioRender.com.

In the spectrum of clinical manifestations characterizing synucleinopathies, sleep disorders are one of the most common complaints. This is due to the existence of comorbidities that are commonly found in elderly people and to disease-specific pathophysiological mechanisms. Brain structures controlling in circadian rhythm, sleep onset, and sleep cycle are susceptible to selective damage due to *α*-syn aggregates accumulation and spreading (Figure 1A). Furthermore, a vicious circle between disrupted sleep and the accumulation of toxic protein aggregates exists, potentially having a critical role in pathological progression in synucleinopathies.

Given the crucial role of restorative sleep in the overall quality of life, as well as the impact of insufficient sleep on both motor and nonmotor symptoms, the management of patients affected by synucleinopathies should also include effective treatments for sleep disorders. In the current landscape of existing pharmacological and non-pharmacological strategies, exercise therapy is particularly appealing. Emerging evidence suggests that physical activity can effectively counteract the neurotoxic effects of α-syn aggregates (Marino et al., 2023). Additionally, the relatively low risk profile associated with patient-tailored exercise programs, makes this approach potentially widely applicable among patients (Amara and Memon, 2018).

A wide variety of studies have explored the benefits of different exercise protocols in clinically manifest PD (Table 1), as well as the potential use of physical activity as part of preemptive strategies to preserve cognitive function and autonomy has also been considered in early forms of synucleinopathy, such as primary RBD (Johnson and Westlake, 2018). With the exploration of new tools for the diagnosis of synucleinopathies at prodromal stages (Stefani et al., 2021; Bellomo et al., 2022), the identification of low-risk disease modifying strategies is gaining even higher relevance.

Despite the growing evidence supporting the benefits of exercise therapy in improving sleep outcomes in synucleinopathies, further research is needed to establish specific guidelines and recommendations for exercise prescription in this population. Longitudinal studies with larger sample sizes are warranted to elucidate the optimal type, intensity, frequency, and duration of exercise required to achieve meaningful improvements in sleep quality and sleep-related outcomes. Additionally, studies investigating the mechanisms underlying the beneficial effects of exercise on circadian rhythms and sleep in synucleinopathies, including its impact on *α*-syn pathology, neurotransmitter systems, and neuroinflammation (Figure 1B), are essential for advancing our understanding and informing clinical practice.

## Footnotes

^1^ https://pubmed.ncbi.nlm.nih.gov/
